# Medical Persecution in British Columbia

**Published:** 1891-07

**Authors:** John Hall

**Affiliations:** Victoria, B. C.


### MEDICAL PERSECUTION IN BRITISH COLUMBIA.

EDITORS OF THE HOMŒOPATHIC PHYSICIAN:

It may be as well that your numerous readers, particularly those seeking new homes for their medical skill, should know the real condition of things in this Province. Indeed, such knowledge ought, perhaps, have been given sooner had not writing been such a labor.

On coming here from Toronto for a warmer climate, in which city the writer had practiced some thirty years, he was surprised to find that this very close community, though in Canada, had formed, by the medical men there existing, a bill, afterward passed as law, by which all resident physicians were admitted and called legally qualified practitioners, taking from their number sufficient to form a Board of Medical Examiners for those who might afterward come to this Province with the object of practicing.

The subscriber applied both in Vancouver and Victoria to the officers of this board for liberty to pursue his profession, but was met by the answer of their impossibility of complying with such a request; that no one, whatever his age or experience, could avoid a personal examination by their board, the conditions of which were the payment, first, of a fee of $100, and, if passed, of a subsequent one of $5 annually as a member of that body.

Of course, it will be readily believed that such an assumption of power was strongly protested against, the writer feeling unwilling to submit, while prerogative was in their hands and the door effectually closed. This Province was then left for the States, where such a liberty as was sought for would not be denied. Unfortunately, when there, the subscriber found himself a stranger, none knowing him or those who had written letters of introduction, and weight of years made it almost impossible to face the current of public opinion and hostility which every new-comer must more or less experience, especially in a profession which was so opposite to that spurious Homœopathy which extensively prevailed. Actuated by such thoughts, steps were again directed to Victoria as presenting the most promising field for a successful homœopathic practice. Having this in view, most of the eminent men of Parliament were seen promptly and the position made fully known to them. Among them were the late Hon. Robert Dunsmuir, a member of the Privy Council, and the late Hon. T. M. Humphreys, of the Opposition, both of whom, with many of their colleagues, whose names need not be mentioned, promised cordial support. On the strength of this, at the next assembling of Parliament, a bill was prepared and introduced by Mr. Anderson, embodying mainly that "the present bill was unfair, as expelling a body of men from the Province whom the public had a right to see admitted ; that these practitioners were not afraid of examination, even if severe, but were specially averse to being forced before a hostile board, who, notwithstanding their frequent protestation to the contrary, were opposed to our progress. We particularly desired independence of their examination, and on that ground we sought a board of our own, when sufficient true men could be admitted to form one." This bill would have passed had not the printer made some mistake in rendering it, causing a delay of three days, whereby the dominant school got knowledge of our intentions and mustered all their resources against us, defeating the measure, though its friends had fought ably to the last for it, Mr. Humphreys being so ill at the time as to be hardly able to attend the discussion.

But the bill was lost to our school, the writer's own claim being admitted, provided the Privy Council were satisfied with his diplomas. Their decision resulted satisfactorily. This law admitted us on the same ground as others, but before an opposing board. And, seeming too liberal, at the next session of the Legislature a bill was introduced by the Hon. Theodore Davy with an amendment to the Medical Act "that hereafter no one should be eligible for examination but those who had received their diplomas from a college or university requiring *three years' study of medical science* AS A HABIT." The writer sent a very strong protest to the government paper here, the *Colonist*, which would probably have killed this bill, but it was so passed, the amendment then becoming law, killing our men at one blow.

Since then, medical men have been elected to the Parliament who will surely give their influence against any new legislation in our behalf.

Such is a condensed view of our affairs in British Columbia, called by our enemies fair and impartial, both schools being recognized on the same terms, and this is so lauded by them that the public is made to believe that such conduct is fair and just to us because the law is on the statute book.

JOHN HALL.

VICTORIA, B. C., April 25th, 1891.

